# Cationic Cyclopropenium-Based Hyper-Crosslinked Polymer Enhanced Polyethylene Oxide Composite Electrolyte for All-Solid-State Li-S Battery

**DOI:** 10.3390/nano11102562

**Published:** 2021-09-29

**Authors:** Shuang Lian, Yu Wang, Haifeng Ji, Xiaojie Zhang, Jingjing Shi, Yi Feng, Xiongwei Qu

**Affiliations:** 1 Hebei Key Laboratory of Functional Polymers, Department of Polymer Materials and Engineering, Hebei University of Technology, 8 Guangrong Street, Tianjin 300130, China; lianshuang8069@163.com (S.L.); whhy0320@163.com (Y.W.); haifengji@sohu.com (H.J.); xwqu@hebut.edu.cn (X.Q.); 2 School of Science, Nantong University, Nantong 226019, Jiangsu, China

**Keywords:** solid-state electrolyte, cyclopropenium cationic-based polymer, polyethylene oxide, Li^+^ conductivity, lithium sulfur battery

## Abstract

The development of solid-state polymer electrolytes is an effective way to overcome the notorious shuttle effect of polysulfides in traditional liquid lithium sulfur batteries. In this paper, cationic cyclopropenium based cross-linked polymer was firstly prepared with the one pot method, and then the counter ion was replaced by TFSI^−^ anion using simple ion replacement. Cationic cyclopropenium hyper-crosslinked polymer (HP) was introduced into a polyethylene oxide (PEO) matrix with the solution casting method to prepare a composite polymer electrolyte membrane. By adding HP@TFSI to the PEO-based electrolyte, the mechanical and electrochemical properties of the solid-state lithium-sulfur batteries were significantly improved. The PEO-20%HP@TFSI electrolyte shows the highest Li^+^ ionic conductivity at 60 °C (4.0 × 10^−4^ S·cm^−1^) and the highest mechanical strength. In the PEO matrix, uniform distribution of HP@TFSI inhibits crystallization and weakens the interaction between each PEO chain. Compared with pure PEO/LiTFSI electrolyte, the PEO-20%HP@TFSI electrolyte shows lower interface resistance and higher interface stability with lithium anode. The lithium sulfur battery based on the PEO-20%HP@TFSI electrolyte shows excellent electrochemical performance, high Coulombic efficiency and high cycle stability. After 500 cycles, the capacity of the lithium-sulfur battery based on PEO-20%HP@TFSI electrolytes keeps approximately 410 mAh·g^−1^ at 1 C, the Coulomb efficiency is close to 100%, and the cycle capacity decay rate is 0.082%.

## 1. Introduction

With the continuous development of modernization and the popularity of all kinds of electronic equipment and electric vehicles, the storage of energy has become an important issue in today’s social development. In order to avoid over exploitation and utilization of natural gas, oil, coal and other fossil fuels, reduce environmental pollution, establish a good ecological cycle, and promote the development of a low-carbon economy and secondary energy, there is an urgency to develop high-density energy storage devices. Increasing attention is being given to energy storage devices with high energy density and low prices [1,2,3,4]. Lithium sulfur (Li-S) batteries have received tremendous attention in recent years due to their high theoretical energy density (2600 Wh·kg^−1^) and large theoretical capacity (1675 mAh·g^−1^) [5,6]. Despite their considerable advantages, the application of Li-S batteries has been hindered by the notorious “shuttle effect”, the volume expansion of the sulfur cathode and poor electrical conductivities of sulfur and solid-state discharging products. These disadvantages result in the loss of active materials and low Coulombic efficiency [7,8,9,10]. Additionally, lithium itself has serious safety hazards. Lithium easily reacts with most electrolytes, especially the liquid organic electrolytes used in batteries. The growth of lithium dendrite will pierce the separator and lead to short circuit of the batteries. Although the additives to conventional liquid organic electrolytes can inhibit the growth of lithium dendrite, they are not a good strategy to solve the safety issues associated with Li-S batteries [11]. As a matter of fact, liquid organic electrolytes are highly flammable and easily ignited in Li-S batteries. The most effective solution is to replace the traditional liquid organic electrolytes with safer electrolytes.

Solid polymer electrolytes (SPEs) have been widely studied due to their high modulus, and are considered to be the most direct way to commercialize high-performance rechargeable batteries [12]. SPEs can build a physical barrier for the growth of lithium dendrite, so it is necessary to develop safer and more reliable solid polymer electrolytes with high mechanical properties. Solid polymer electrolytes have the advantages of easy synthesis, low energy density, high modulus and low cost, which have all fueled interest in SPEs [13]. Polyethylene oxide (PEO) is a common kind of solid polymer electrolyte, but it suffers from high crystallinity at room temperature and the poor mechanical properties [14,15]. To solve this issue, many approaches have been attempted by adding inert fillers, such as Al_2_O_3_ [16], SiO_2_ [17], Fe_3_O_4_ [18], and active fillers such as Li_7_La_3_Zr_2_O_12_(LLZO) [19], Li_10_SIP_2_S_12_(LSPS) [20] and Li_10_GeP_2_S_12_(LGPS) [21] into a PEO matrix for achieving lower crystallinity and higher ionic conductivity.

In recent years, covalent organic polymers (COPs) have shown great potential in gas separation, energy storage, electronic devices, and other fields due to their controllable and stable structures [22,23,24]. Cationic organic polymers are a class of COPs that have been widely studied. According to Guo’s report, the synergistic effect of ionic COPs and PEG contributes to the preparation of solid-state electrolytes with high ionic conductivity [25]. For solid state electrolytes, Li^+^ and related anionic species combine to form ion pairs or aggregates under the strong Coulomb effect [26]. It is reported that cationic COPs can effectively break the Coulomb interaction due to its high polarizability, dissociation ion pairs or aggregates of lithium salt, increasing the concentration of free moving Li^+^ and improvement of its conductivity [26]. However, there are few studies on aromatic cationic COPs.

Cyclopropane cations are the smallest aromatic structures with a positive charge. The stability of the triangular skeleton can be further enhanced by introducing amino groups through SN_2_ reactions. The physical and chemical properties of cyclopropane cations are obtained by substitutes [27]. Therefore, they are used as ionic liquids, redox active polymers, and other bioactive compounds [28,29,30]. Our previous work has shown that the cyclopropenium cationic-based polymer electrolyte possesses high conductivity and lower crystallinity [31]. However, the rigid skeleton limits the further improvement of the doping ratio. Considering the poor mechanical properties of inorganic fillers and the restraint mentioned above, herein, we demonstrate a facile method toward the aromatic cyclopropane polymer. We used a symmetrical double six membered cyclic amino small molecule and pentachlorocyclopropane to prepare HP@Cl. HP@TFSI was obtained by replacing Cl^−^ with TFSI^−^. The carbon flexible chain structure was introduced into the polymer skeleton to reduce the rigidity of cyclopropenium cationic polymer. A PEO-based polymer electrolyte was applied to a lithium sulfur battery. The lithium sulfur battery with PEO-20%HP@TFSI electrolyte has excellent electrochemical performance, better coulomb efficiency and excellent cycle stability.

## 2. Materials and Methods

### 2.1. Material

The purity of the chemical reagents used are at least analytical grade. Pentachlorocyclopropane (61 USD/100 g) was produced by Saen Chemical Technology Co., Ltd., (Shanghai, China). 1,3-Bis-(4-piperidyl)propane (87 USD/100 g), triethylamine, poly(ethylene oxide) (*M_w_* = 600,000, 99.9%) (56 USD/100 g) and LiTFSI (99.9%) (61 USD/100 g) were purchased from Aladdin reagent Co., Ltd., (Shanghai, China). Chloroform, dichloromethane and ethanol were purchased from Fuchen Chemical Reagent Co., Ltd. (Tianjin, China).

### 2.2. Fabrication of HP@Cl and HP@TFSI

A 50 mL round bottomed flask was vacuumized and then filled with dry nitrogen. Pentachlorocyclopropane (500 mg, 2.3 mmol), 1,3-bis (4-piperidinyl) propane (736 mg, 3.5 mmol) and triethylamine (590 mg, 5.8 mmol) were dissolved in 15 mL anhydrous chloroform, and stirred at room temperature for 12 h. At the end of the reaction, a large amount of dichloromethane and distilled water were used to wash the solid product at least three times. The unreacted raw materials were removed by stirring in distilled water for 24 h at room temperature, and then dried under 60 °C vacuum for 24 h to obtain yellow solid powder (HP@Cl) (yield 88%). Next, HP@Cl was put in the aqueous solution of LiTFSI, stirred for 24 h at room temperature, repeating the operation twice. Finally, the yellow powder was dried for 12 h under a 60 °C vacuum circumstance to obtain HP@TFSI.

### 2.3. Preparation of Sulfur Composite and Electrode

S and Super-P were fully ground at a mass ratio of 60:40 for 1 h. Then, the mixture was sealed in a PTFE hydrothermal reactor (Xi’an YIBEIER Equipment Company, Xi’an, Shanxi, China) under argon atmosphere and heated at 155 °C to obtain S@C composite. The S@C composite and polyvinylidene fluoride (PVDF) were mixed and ground at the mass ratio of 9:1 for 1 h. Next, a slurry was obtained by adding N-methyl-2-pyridinone (NMP) into the mixture powder. The slurry was then coated on the aluminum foil electrode. The electrode was used after drying at 60 °C for 12 h. The dried electrode was cut into a disc with a diameter of 10 mm for assembling the battery. The sulfur load per unit area was 0.8 mg·cm^−2^.

### 2.4. Batteries Assembling Steps

The solid state Li-S batteries were assembled using a CR-2032 type coin cell (Guangdong Canrd New Energy Technology Co.,Ltd, Dongguan, Guangdong, China) in a glove box filled with argon (99.9995% purity). The cell was stacked with cathode cell cap, S@C composite electrode, polymer electrolyte membrane, lithium metal and anode cell cap. The prepared electrolyte serves as both the electrolyte and the separator [32].

## 3. Results and Discussion

**Figure 1:**
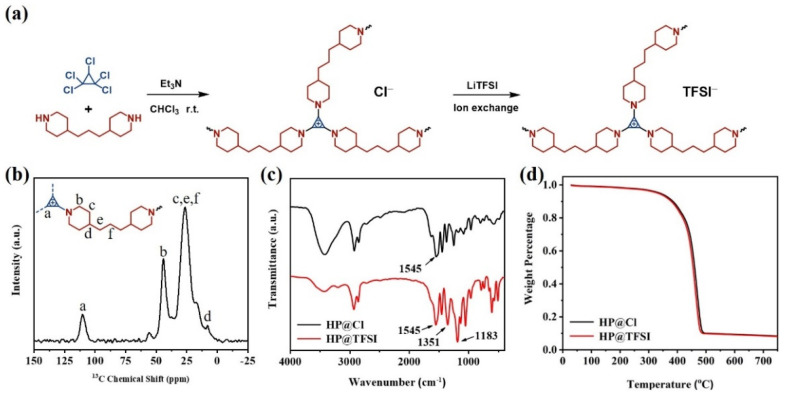
(**a**) Preparation of HP@X; (**b**) Solid-state NMR of HP@Cl; (**c**) FT-IR spectra of HP@Cl and HP@TFSI; (**d**) TGA curves of HP@Cl and HP@TFSI.

The preparation sketch of cyclopropenium cationic-based HP is shown in Figure 1a, and the related SN_2_ mechanism is shown in Scheme S1. Bis (trifluoromethylsulfonyl) imide anions (TFSI^−^) have been reported to have higher conductivity than other weak anions, such as tetrafluoroborate (BF_4_^−^) and hexafluorophosphate (PF_6_^−^), because of the plasticizing effect of -SO_2_-N-SO_2_- in TFSI^−^ [14]. So TFSI^−^ was selected as the final counter ion through a simple ion exchange process. The HP@Cl structure was clearly observed from three ^13^C resonance peaks centered at 110, 44, 26 ppm, corresponding to the cyclopropenium ring, tertiary carbon, and methylene carbon in the HP@Cl (Figure 1b). In the FT-IR spectrum of HP@Cl (Figure S1), the stretching vibration peak of the C-Cl bond in pentachlorocyclopropane at 635 cm^−1^ disappeared, and a signal at 1545 cm^−1^ corresponding to the tensile vibration peak of the aromatic cyclopropenium ring was observed, which proves that the cationic-based HP were successfully obtained. Peaks were observed at 3433 cm^−1^, corresponding to O-H stretching vibrations of HP@Cl. The Cl^-^ ion has strong hydrophilicity and it is easy to combine with water vapor in air through hydrogen bond. Through simple ion exchange, we prepared the HP@TFSI. The absorption peaks of the two cyclopropenium cationic polymers are shown in Figure 1c. The stretching vibration peak of SO_2_ (at 1351 cm^−1^) and CF_3_ groups (at 1183 cm^−1^) can be clearly observed, which proves that Cl^−^ was successfully replaced by TFSI^−^. In addition, the TGA curves revealed that both HP@Cl and HP@TFSI possess high thermal stability with a decomposition temperature up to 300 °C (Figure 1d). It is likely to meet the requirements of energy storage equipment.

In order to study the crystallinity of cyclopropenium cationic polymer (HP@Cl and HP@TFSI), an X-ray diffraction (XRD) test was carried out. As shown in Figure S2, it can be observed that there is no diffraction peak in the range of 0.5~10° for the two cyclopropenium cationic polymers, which proves that there is no regular pore structure in either HP@Cl and HP@TFSI. But there is an obvious broad diffraction peak in the range of 5~80°, which indicates that the structure is a stable amorphous phase.

**Figure 2:**
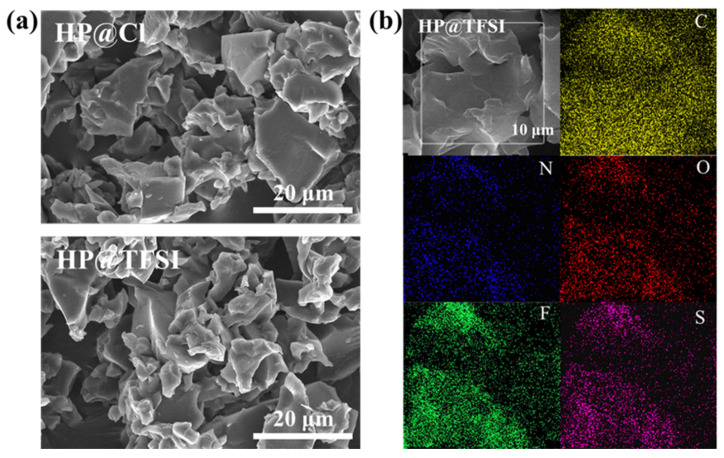
SEM images of (**a**) HP@Cl and HP@TFSI; (**b**) EDS mapping of HP@TFSI.

The particle morphology of the two kinds of cyclopropenium cationic polymers was characterized by the SEM and EDS. As shown in Figure 2a, there are irregular blocks without obvious pore structure in two cationic polymers, which is consistent with the result of PXRD. From the EDS diagram in Figure 2b, it can be clearly observed that the elements C, N, O, F and S are well dispersed in HP@TFSI. It is further proved that there were no Cl^−^ ions in the particles and TFSI^−^ ions were successfully exchanged, which is significantly helpful for the improvement of the conductivity of PEO/LiTFSI/HP@TFSI electrolytes.

**Figure 3:**
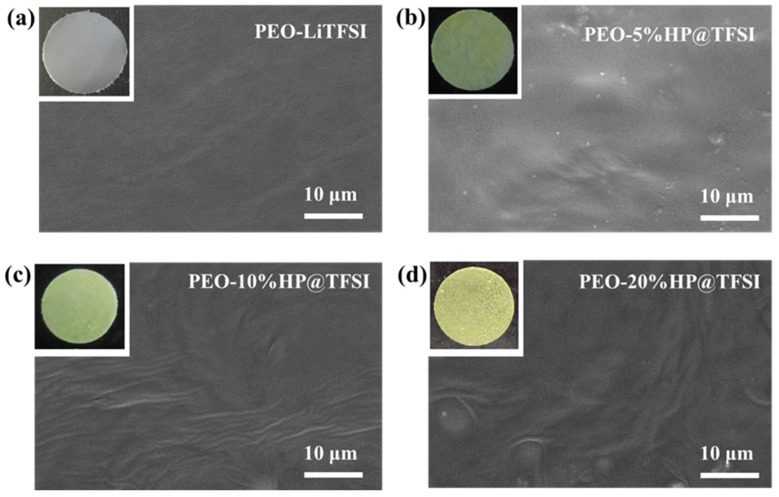
Photos and SEM images of (**a**) PEO-LiTFSI; (**b**) PEO-5%HP@TFSI; (**c**) PEO-10%HP@TFSI; (**d**) PEO-20%HP@TFSI membranes.

HP@TFSI were dispersed into PEO-LiTFSI acetonitrile solution followed by solvent elimination to obtain the SPEs. The scatter uniformity of fillers affects the electrochemical performance of polymer electrolyte. In order to observe the micro morphology of polymer electrolytes, the polymer electrolytes were tested by SEM. From the SEM images in Figure 3a–d, it can be clearly observed that the surface of the composite polymer electrolytes is still smooth, which is formed by incorporating 5% to 20%HP@TFSI into the PEO electrolytes. With the increase of HP@TFSI addition, there is no aggregation phenomenon in the polymer electrolytes. The uniformity of the HP@TFSI distribution was revealed by SEM elemental mapping, as shown in Figure S3, and it is also confirmed that those HP@TFSI particles dispersed very well in the PEO, and that the HP@TFSI particles cannot be seen on the surface of polymer electrolytes with 20 wt% addition. The C, N, O, F and S elements in the polymer electrolyte were uniformly distributed in the whole PEO matrix, which is conducive to the improvement of the performance of PEO based electrolyte.

**Figure 4:**
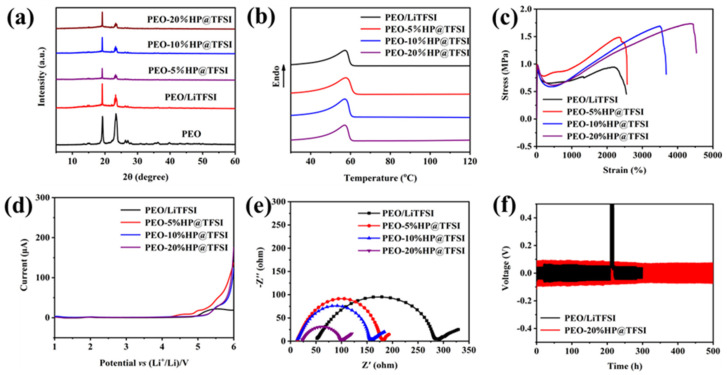
(**a**) XRD patterns of PEO and PEO-HP@TFSI polymer electrolytes; (**b**) DSC traces of PEO and PEO-HP@TFSI polymer electrolyte; (**c**) Tensile stress-strain curve of polymer electrolytes; (**d**) LSV curves of polymer electrolytes at 60 °C; (**e**) Interface EIS spectrum of polymer electrolytes; (**f**) Li plating and stripping test at a current density of 0.1 mA·cm^−2^ at 60 °C.

The X-ray diffraction patterns (XRD) of PEO and electrolytes with different HP@TFSI content were analyzed. It can be seen from Figure 4a that there are two obvious peaks in pure PEO phase at 19° and 23° corresponding to (120) and (112) crystal faces respectively [33]. With the addition of HP@TFSI, the decrease of peak intensity of the PEO/LiTFSI/HP@TFSI electrolyte indicates the increase of amorphous region in PEO. This is because with the increase of LiTFSI and HP@TFSI, the regular chain structure of PEO is destroyed.

The DSC test is shown in Figure 4b. PEO is a crystalline phase, and there are ordered molecular chains inside the polymer. The addition of fillers will destroy the molecular chain arrangement, resulting in the decrease of crystallinity. With the increase of the amount of HP@THSI, the crystallinity of PEO-20%HP@TFSI is the lowest. Li^+^ transportation mainly occurs in the amorphous part of PEO, so the decrease of crystallinity of PEO is beneficial to improve the conductivity of the electrolytes. The HP@TFSI addition of PEO can also inhibit the crystallization of the polymer and increase the number of amorphous regions in the polymer electrolyte, which can further promote the migration of Li^+^ in the polymer network. The detailed crystallinity was calculated using equation [34]

(1) $$\chi_{c}=\frac{\Delta H_{m}}{\Delta H_{PEO}f_{PEO}}$$

in which Δ*H*_m_ is the melting enthalpy of SPEs, Δ*H*_PEO_ is 196.4 J·g^−1^, and $f_{PEO}$ represents the PEO mass fraction. PEO-20%HP@TFSI exhibits the lowest *T*_m_ (56.3 °C) and $\chi_{c}$ (36.6%) (Table 1).

A polymer electrolyte membrane with strong mechanical properties can resist the deformation of the battery during assembly and cycling, thus improving the safety performance of Li-S batteries. The stress-strain curve of polymer electrolyte membranes was analyzed, as shown in Figure 4c, and the data are summarized in Table S2. The mechanical properties of the polymer electrolytes are enhanced with an increased amount of HP@TFSI. The tensile strength of the PEO-20%HP@TFSI electrolytes increases from 0.95 to 1.45 MPa, and the elongation at break increases from 2549.4% to 4533.82%. The enhanced mechanical properties of the PEO/LiTFSI/HP@TFSI electrolytes can be ascribed to the framework support of cationic-based COP. The dispersion of HP@TFSI particles greatly reduces the stress between PEO chains, resulting in improved toughness of PEO chains.

Li^+^ conductivity is the most significant parameter to assess the performance of SPEs. The relationship between ionic conductivity and temperature are shown in Figure 4d. The ionic conductivity can be calculated by equation

(2) $$\sigma =\frac{L}{RS}$$

where *L* is the thickness of the electrolyte, *S* is the area of the electrolyte membrane, and *R* is the resistance of the membrane.

The results of ionic conductivity of polymer electrolytes at different temperatures are shown in Table S1. It can be seen from the table that the ionic conductivity of all polymer electrolytes has a significant increase above 50 °C, which corresponds to the transition of PEO to molten state. The ionic conductivity of PEO-HP@TFSI electrolytes were improved, and the PEO-20%HP@TFSI electrolytes showed the highest ionic conductivity at 60 °C. The transportation of Li^+^ depends on the amorphous phase of PEO matrix. HP@TFSI additives can destroy the ordered molecular structure of PEO, reduce the crystallinity of PEO, and provide more amorphous regions for Li^+^ conduction. The ionic conductivity is comparable to that of the PEO-based electrolytes prepared by adding various fillers as given in previous works (Table S3). Compared to the most reported electrolytes, the PEO-20%HP@TFSI electrolyte has higher ionic conductivity, Li^+^ transference, and cycling performance.

The EIS spectrum of the interface of the symmetrical battery based on polymer electrolyte is shown in Figure 4e. The unstable interface leads to poor electrochemical performance during the cycle. The data are summarized in Table 2. In the four kinds of polymer electrolytes, with an increase in temperature and HP@TFSI, the interfacial impedance of electrolyte decreases. The cell with PEO-20%HP@TFSI electrolyte has the lowest resistance 77.6 Ω. The results show that the pores of PEO/LiTFSI electrolytes filled with HP@TFSI particles can effectively increase the contact area between the lithium electrode and polymer electrolytes and improve the interface stability of all solid-state Li-S batteries.

To further test the interface stability between PEO-20%HP@TFSI electrolytes and lithium electrodes, a symmetrical battery was tested at 60 °C. Results are shown in Figure 4f. The symmetrical battery with PEO-20%HP@TFSI electrolytes can cycle for 500 h and maintain a relatively stable polarization voltage of about 80 mV. The strong cycle stability of the PEO-20%HP@TFSI electrolytes to lithium is due to its better mechanical properties and lower impedance, which is conducive to the improvement of the performance of all-solid-state Li-S batteries.

Besides the ionic conductivity, the Li^+^ transference number ($t_{Li^{+}}$) can also be used to evaluate polymer electrolytes. The chronoamperometry and EIS spectra of four kinds of polymer electrolyte lithium symmetric batteries at 60 °C are shown in Figure S5. The Li^+^ migration number is calculated by the equation

(3) $$t_{Li^{+}}=\frac{I_{s}\left(\Delta V-I_{0}R_{0}\right)}{I_{0}\left(\Delta V-I_{S}R_{S}\right)}$$

where ΔV is 0.01 V, $R_{0}$ and $R_{S}$ are the initial and steady-state interface impedance, respectively. And $I_{0}$ and $I_{s}$ are the initial and steady-state current, respectively [35]. The value of $t_{Li^{+}}$ in Table 2 was calculated from the data shown in Figure S6 using equation 3. As can be seen from the Table 2, with an increase of HP@TFSI particle, the Li^+^ migration number of polymer electrolytes increases. In particular, when the addition amount is 20 wt%, the Li^+^ migration number of polymer electrolyte is as high as 0.521, which proves that more than half of Li^+^ can pass through the electrolyte membrane. The increase in Li^+^ migration number can be explained by the Lewis acid–base theory: the oxygen atoms (Lewis base sites) are closely associated with the cationic cyclopropenium components (Lewis acid sites) in HP@TFSI, thus weakening the combination between the oxygen atoms and the Li^+^ [36]. In addition, the increase in amorphous regions of the polymer can also accelerate the shuttling of Li^+^.

The thermal analysis of PEO and polymer electrolytes was investigated, and the result is shown in Figure S7. Due to the water absorption of the polymer electrolytes, the water in the air will be absorbed during the test, resulting in a weight loss of about 4% before 100 °C in the thermogravimetric curve. The decomposition temperature of the four polymer electrolytes is higher than 300 °C, which meets the requirements of general energy storage equipment.

In addition, the electrolyte membrane is sandwiched between the stainless steel and the lithium sheet to form an asymmetric battery for measuring the electrochemical window, as shown in Figure S4. It can be seen that the PEO-20%HP@TFSI polymer electrolyte shows high oxidation potential, which can meet the actual voltage demand of a Li-S battery.

**Figure 5:**
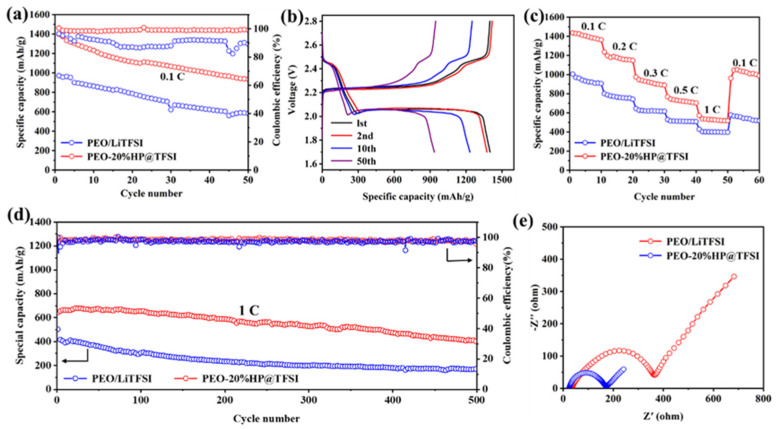
(**a**) cycle performance; (**b**) charge-discharge curves; (**c**) rate performance; (**d**) long-term cyclic stability of PEO-based battery at 1 C (60 °C); (**e**) AC impedance spectra of lithium sulfur battery based on PEO/LiTFSI and PEO-20%HP@TFSI at 60 °C.

In order to study the electrochemical performance of polymer electrolytes in Li-S batteries, the cycling capacity of a Li-S battery based on solid electrolytes was tested at 60 °C at a current rate of 0.1 C. The results are shown in Figure 5a. The initial discharge specific capacity of the cell with PEO-20%HP@TFSI electrolytes was 1400 mAh·g^−1^ and the Coulomb efficiency was close to 100%, while the initial discharge specific capacity of battery with PEO/LiTFSI electrolyte was 972 mAh·g^−1^ and the Coulomb efficiency was unstable. The test results show that the battery based on PEO-20%HP@TFSI electrolyte had stable cycle performance. As shown in Figure S8, the CV Curve of Li-S battery with PEO-20%HP@TFSI electrolyte was between 1.7 V and 2.8 V. Two platforms can be observed (Figure 5b). The first platform is at 2.4 V, corresponding to the reduction of S_8_ to lithium polysulfide (Li_2_S_n_, 2 < n < 8), and the second long platform is at 2.0 V, corresponding to the conversion of lithium polysulfide to Li_2_S_2_ or Li_2_S. The typical redox peak of a lithium sulfur battery can be observed on the CV curve. It can be seen that the first three cycles almost overlap, indicating that the electrochemical polarization of all solid-state Li-S batteries with PEO-20%HP@TFSI electrolytes is low. The rate performance of all solid-state lithium sulfur batteries are presented in Figure 5c. The specific capacities of the first cycle are 1435, 1235, 974, 768, 572 mAh·g^−1^ at 0.1 C, 0.2 C, 0.3 C, 0.5 C, 1 C, respectively. In addition, the corresponding charging and discharging curves at different rates are shown in Figure S9. With an increase in rate, the discharge platform of the electrode becomes shorter and shorter. The discharge platform can be clearly observed even at high current density (1 C), which indicates that the PEO-20%HP@TFSI electrolyte has good stability. When the discharge current density reverted back to 0.1 C, it still delivered a highly reversible capacity of 953 mAh·g^−1^. As shown in Figure 5d, the batteries were tested under 1 C high current density to evaluate their structural stability. The corresponding energy density cycle curve was shown in Figure S10. The batteries based on the PEO-20%HP@TFSI electrolytes show better cycle stability and Coulombic efficiency and retain a capacity of 410 mAh·g^−1^ after 500 cycles with a 0.082% decay rate for each cycle. The Coulombic efficiency was above 95% during the whole cycle, whereas the PEO/LiTFSI electrolytes showed severe overcharge with a 0.119% decay rate per cycle.

The lithium-sulfur battery with PEO-20%HP@TFSI electrolytes showed excellent cycle performance and stable Coulomb efficiency. The AC impedance spectra of the lithium sulfur battery assembled with PEO/LiTFSI and PEO-20%HP@TFSI electrolytes are shown in Figure 5e. The Nyquist diagrams of the two lithium-sulfur batteries are semicircle in the high frequency part and inclined in a straight line in the low frequency part. The semicircle corresponds to the charge transfer resistance (RCT) [36] between the electrode and the electrolyte. The experimental results show that because of the stable solid electrolyte interface (SEI), the cell with a PEO-20%HP@TFSI electrolyte membrane shows relatively low interface resistance.

## 4. Conclusions

In summary, cyclopropenium cationic covalent organic polymers with flexible structures were designed, and synthesized solid PEO/TFSI electrolytes with different HP@TFSI contents. As the filler of PEO based electrolyte, flexible structure polymers not only increased the doping amount (20 wt%) and reduced the crystallinity of PEO, but also improved the mechanical properties of the polymer electrolyte. The PEO-20%HP@TFSI electrolytes showed the highest ionic conductivity (4.0 × 10^−4^ S·cm^−1^) and lithium-ion migration number (0.521), and the best mechanical properties (tensile modulus and elongation at break are 1.45 MPa and 4533.8%, respectively). All solid-state lithium-sulfur batteries with PEO-20%HP@TFSI electrolytes had stable Coulomb efficiency and excellent cycle performance. At a current density of 0.1 C, the initial specific capacity of the lithium-sulfur battery was 1400 mAh·g^−1^. At a current density of 1 C, the specific capacity of the battery can be maintained at 410 mAh·g^−1^ after 500 cycles. In general, we believe that cationic COP will become a potential candidate for PEO additives due to its low cost and good structural designability, even though it still has some problems in commercialization, such as toxic intermediates and low production capacity.

## Figures and Tables

**Table 1:** DSC results of polymer electrolyte.

Electrolytes	*T*_m_/°C	Δ*H*_m_/J·g^−1^	$\chi_{c}/\%$
PEO-LiTFSI	57.5	60.25	40.6
PEO-5%HP@TFSI	57.2	56.82	40.2
PEO-10%HP@TFSI	56.9	55.15	38.6
PEO-20%HP@TFSI	56.3	45.3	36.6

**Table 2:** Li^+^ ion transference number data of polymer electrolytes.

Electrolyte	*I*_0_/mA	*I*_s_/mA	*R*_0_/Ω	*R*_s_/Ω	Δ*V*/mV	$t_{{\mathrm{Li}}^{+}}$
PEO/LiTFSI	0.036	0.021	236.4	248.1	10	0.183
PEO-5%HP@TFSI	0.036	0.020	146.2	159.4	10	0.287
PEO-10%HP@TFSI	0.035	0.015	135.3	168.7	10	0.319
PEO-20%HP@TFSI	0.042	0.024	77.6	108.1	10	0.521

## Data Availability

The data presented in this study are available in the main manuscript and in the Supplementary Materials.

## Supplementary Materials

The following are available online at https://www.mdpi.com/article/10.3390/nano11102562/s1, Figure S1: FT-IR spectra of pentachlorocyclopropane, 1,3-Bis-(4-piperidyl)propane and HP@Cl. Figure S2: Powder X-ray diffraction (PXRD) of HP@Cl and HP@TFSI. Figure S3: SEM image and EDS mapping of (a) PEO-5%HP@TFSI, (b) PEO-10%HP@TFSI and (c) PEO-20%HP@TFSI. Figure S4: Curves of polymer electrolyte ionic conductivity with temperature. Figure S5: Chronoamperometric curves and EIS spectrum of lithium symmetrical battery based on (a) PEO/LiTFSI, (b) PEO-5%HP@TFSI, (c) PEO-10%HP@TFSI and (d) PEO-20%HP@TFSI electrolyte at 60 °C. Figure S6: EIS spectra of (a) PEO/LiTFSI, (b) PEO-5%HP@TFSI, (c) PEO-10%HP@TFSI and (d) PEO-20%HP@TFSI. Figure S7: TGA curves of polymer electrolyte. Figure S8: Cyclic voltammetry curve measured at 60 °C based on PEO-20%HP@TFSI electrolyte lithium-sulfur battery. Figure S9: The charge-discharge curves of S@Super P|PEO-20%HP@TFSI|Li at 60 °C at different rates. Figure S10: Energy density performance of PEO-based battery at 1 C (60 °C). Scheme S1: Mechanism of the reaction. Table S1: Ionic conductivity results of polymer electrolyte (Unit: S·cm^−1^). Table S2: Data of mechanical properties number of the four polymer electrolytes. Table S3: Datas of Li-S batteries using different type of PEO-based electrolytes reported in literature.
