# Supplementary material for: Cationic Cyclopropenium-Based Hyper-Crosslinked Polymer Enhanced Polyethylene Oxide Composite Electrolyte for All-Solid-State Li-S Battery
Source: Nanomaterials (Basel). 2021 Sep 29;11(10):2562. doi: 10.3390/nano11102562 (PMC8540722; doi:10.3390/nano11102562)
Supplement: Supplementary file 1 [file nanomaterials-11-02562-s001.zip › nanomaterials-1368553-supplementary.pdf]

nanomaterials

Supporting information

Cationic Cyclopropenium-Based Hyper-Crosslinked Polymer Enhanced Polyethylene Oxide Composite Electrolyte for All-Solid-State Li-S Battery

Shuang Lian [1], Yu Wang [1], Haifeng Ji [1], Xiaojie Zhang [1,*], Jingjing Shi [2,*], Yi Feng [1,*] and Xiongwei Qu [1]

[1] Hebei Key Laboratory of Functional Polymers, Department of Polymer Materials and Engineering, Hebei University of Technology, 8 Guangrong Street, Tianjin 300130, China; lianshuang8069@163.com (S.L.); whhy0320@163.com (Y.W.); haifengji@sohu.com (H.J.); xwqu@hebut.edu.cn (X.Q.)
[2] School of Science, Nantong University, Nantong 226019, Jiangsu, China
* Correspondence: zhangxj@hebut.edu.cn (X.Z.); shijingjing@ntu.edu.cn (J.S.); luckyii0512@163.com (Y.F.); Tel.: +86-13843143643 (X.Z.)

Characterization

The samples were characterized by Vector-22 Fourier transform infrared (FT-IR) spectrometer from Brucker, Germany. Solid-state nuclear magnetic resonance (NMR).

spectra were acquired on an Agilent 600 M (Agilent, USA). The X-ray powder diffraction (XRD) pattern was characterized using the D8 advanced system (Bruker, Germany), equipped with a Cu-Kα radiation source at the scanning rate of 6°/min within 2θ angular range from 5° to 65°. The morphology and structure of the samples were observed by a Nova Nano SEM 450 scanning electron microscopy (FEI, USA). The thermal stability was tested on a TG/DTA6300 (Hitachi, JPN), and the heating rate from ambient temperature to 800 °C was 10 °C·min^{-1} under N_2 gas flow. The crystallinity of the sample was investigated by a diamond DSC differential scanning calorimeter from Perkin Elmer, the United State, and the heating rate from −20 to 150 °C was 10 °C·min^{-1}. The mechanical properties of the electrolyte membranes were detected by an electronic universal testing machine controlled by a CMT6104 microcomputer. The test temperature is 25 °C and the speed rate is 1.66 mm·min^{-1}.

Electrochemical Measurements

The electrochemical tests on SPEs were analyzed by a CHI660E electrochemical workstation, Wuhan. The ionic conductivity was tested by EIS in the frequency range from 100,000 to 0.01 Hz at a temperature of 30–90 °C. The Li-ion transference number (t_{Li^+}) of the SPEs is measured through the lithium symmetric cell (Li|SPE|Li) using the combined ac impedance/dc polarization method at 60 °C. Linear sweep voltammetry (LSV) was used to measure the electrochemical window of the electrolyte using a SS|SPE|Li cell with the voltage of 0–6 V at 0.1 mV·s^{-1} and 60 °C. The batteries were assembled in the Ar-filled glovebox using the 2032-type coin cell. The constant current cycle test was carried out on the multichannel battery test system (BST-5V5mA, Neware) with a voltage range of 1.7–2.8 V.

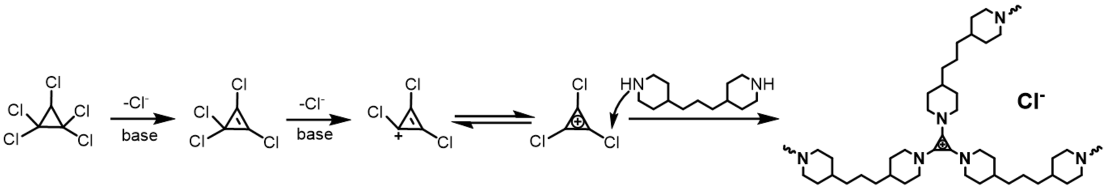

Scheme S1. Mechanism of the reaction.

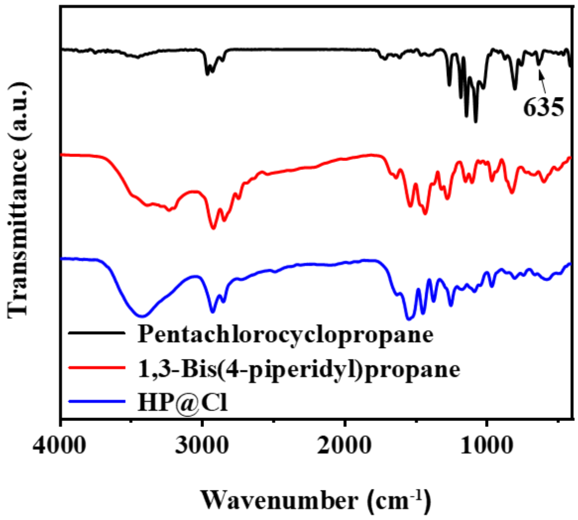

Figure S1. FT-IR spectra of pentachlorocyclopropane, 1,3-Bis-(4-piperidyl)propane and HP@Cl.

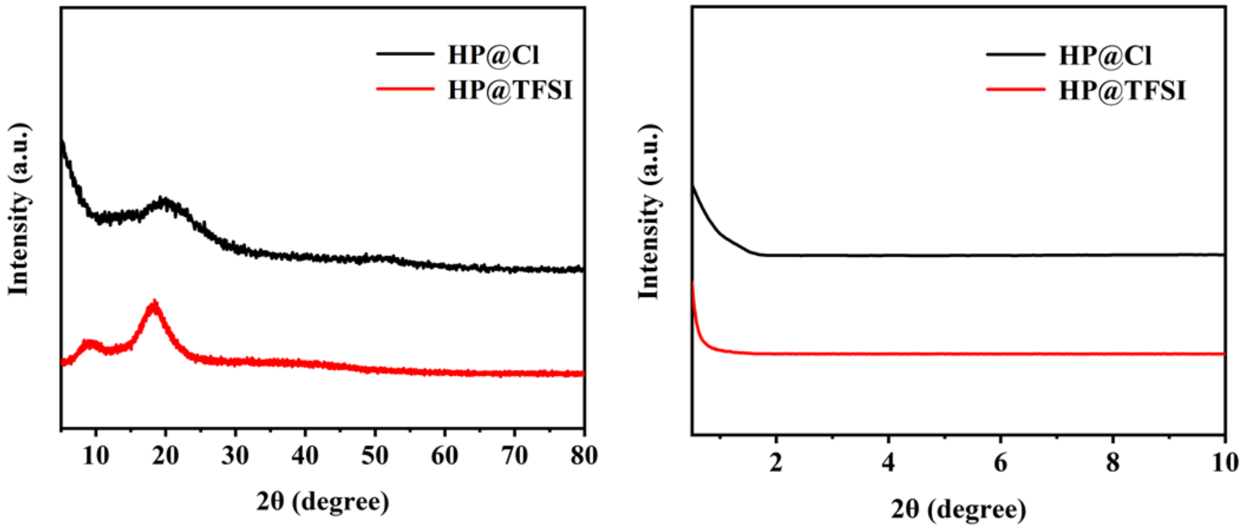

Figure S2. Powder X-ray diffraction (PXRD) of HP@Cl and HP@TFSI.

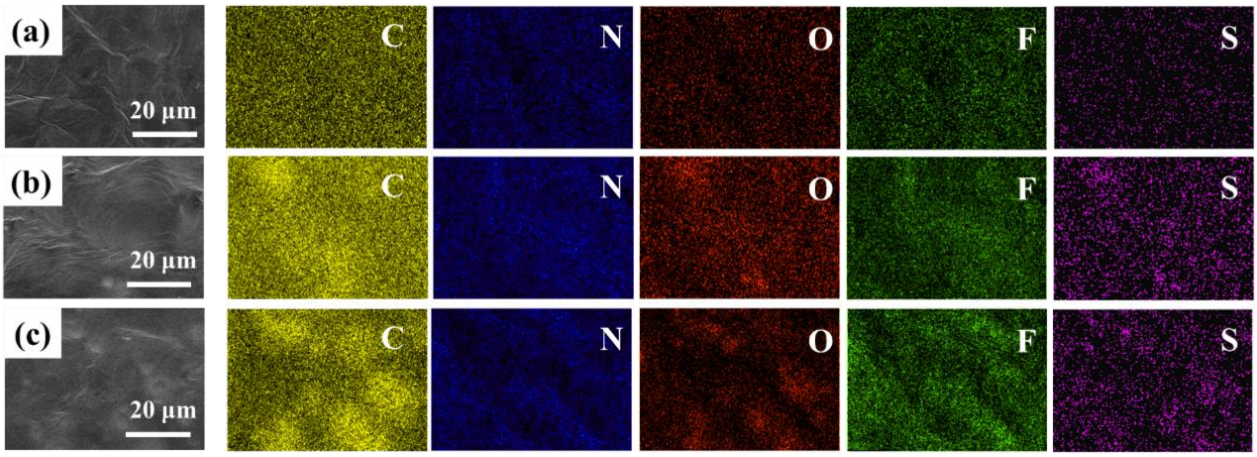

Figure S3. SEM image and EDS mapping of (**a**) PEO-5%HP@TFSI, (**b**)PEO-10%HP@TFSI and (**c**) PEO-20%HP@TFSI.

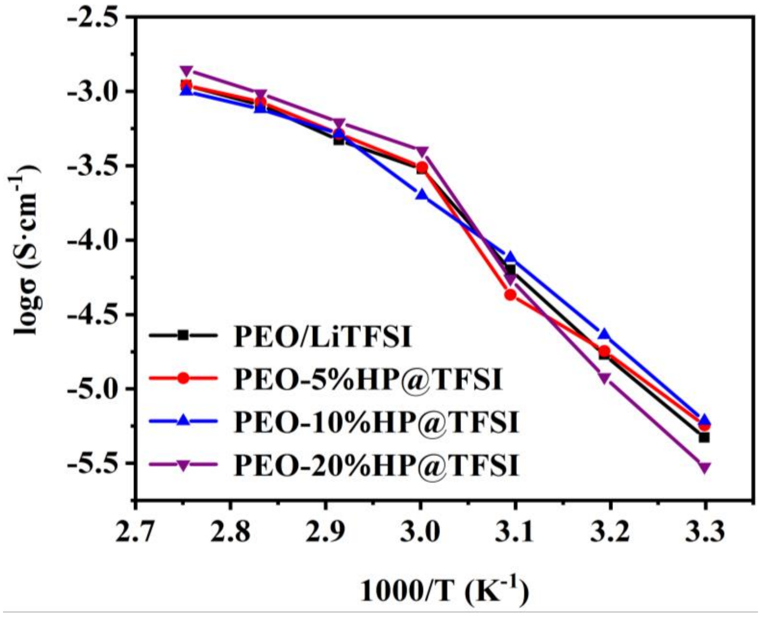

Figure S4. Curves of polymer electrolyte ionic conductivity with temperature.

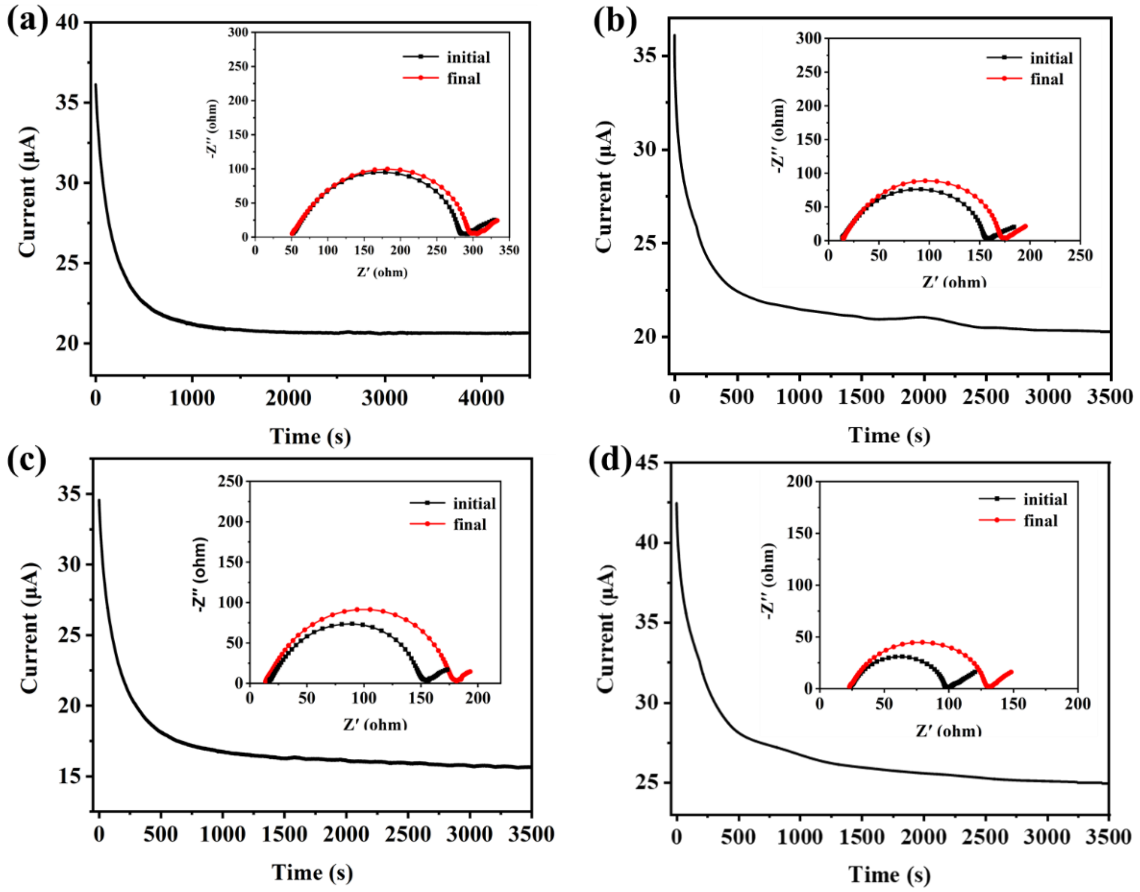

Figure S5. Chronoamperometric curves and EIS spectrum of lithium symmetrical battery based on (**a**) PEO/LiTFSI, (**b**) PEO-5%HP@TFSI, (**c**) PEO-10%HP@TFSI and (**d**) PEO-20%HP@TFSI electrolyte at 60 °C.

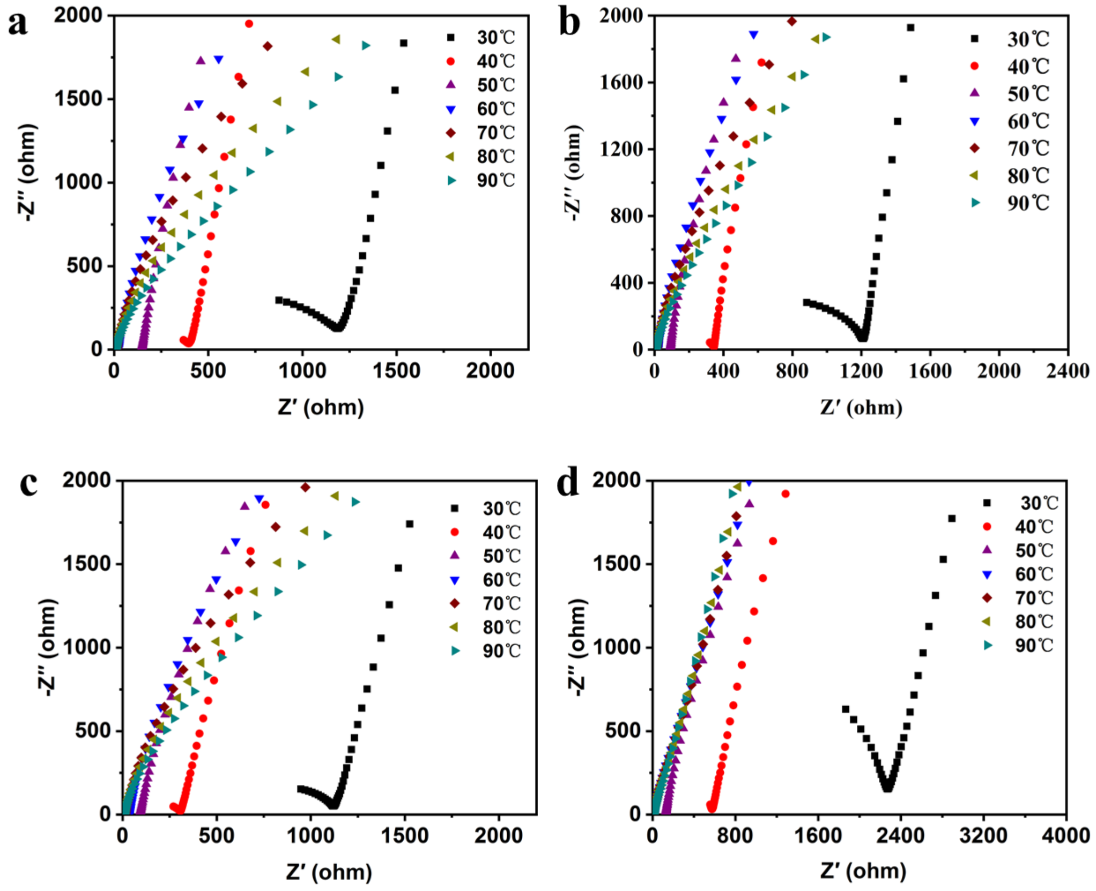

Figure S6. EIS spectra of (**a**) PEO/LiTFSI, (**b**) PEO-5%HP@TFSI, (**c**) PEO-10%HP@TFSI and (**d**) PEO-20%HP@TFSI.

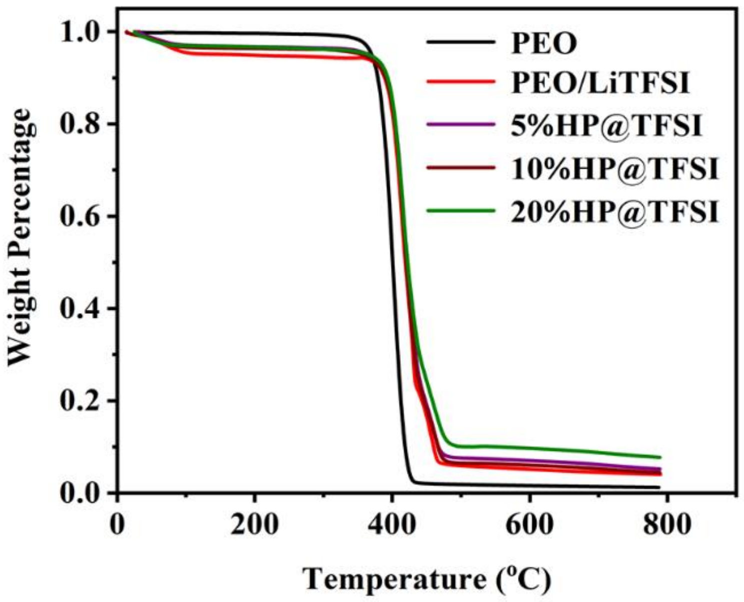

Figure S7. TGA curves of polymer electrolyte.

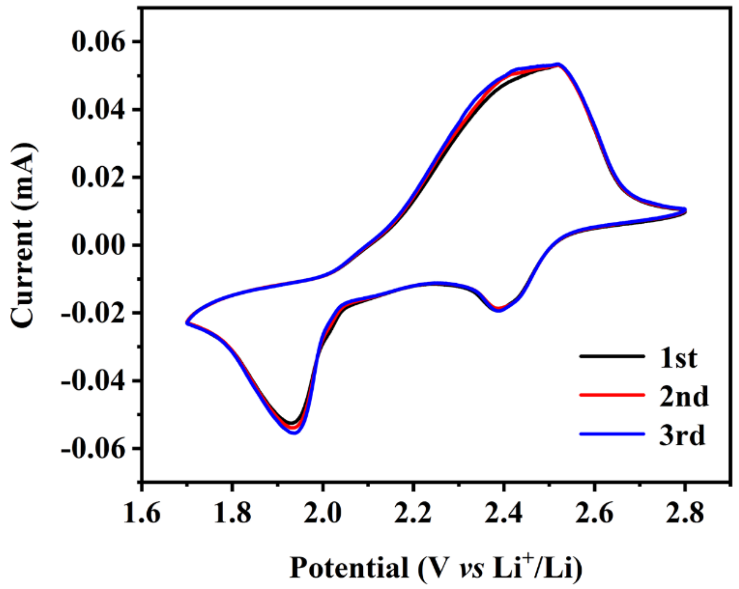

Figure S8. Cyclic voltammetry curve measured at 60 °C based on PEO-20%HP@TFSI electrolyte lithium-sulfur battery.

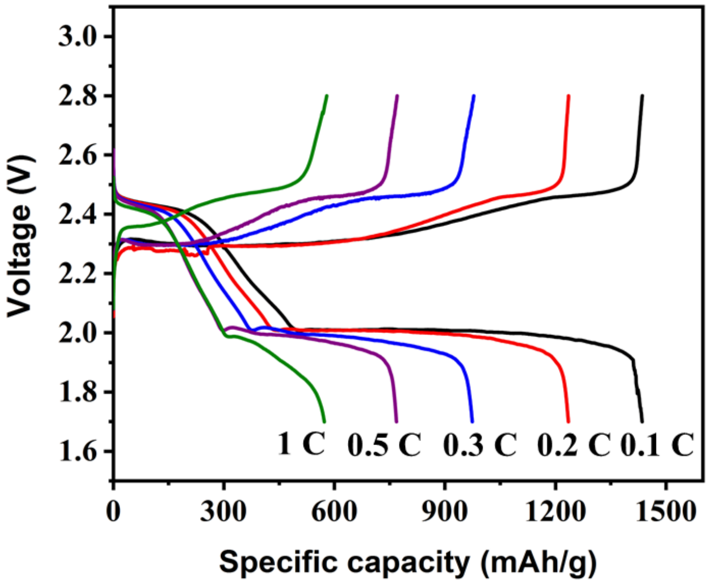

Figure S9. The charge-discharge curves of S@Super P|PEO-20%HP@TFSI|Li at 60 °C at different rates.

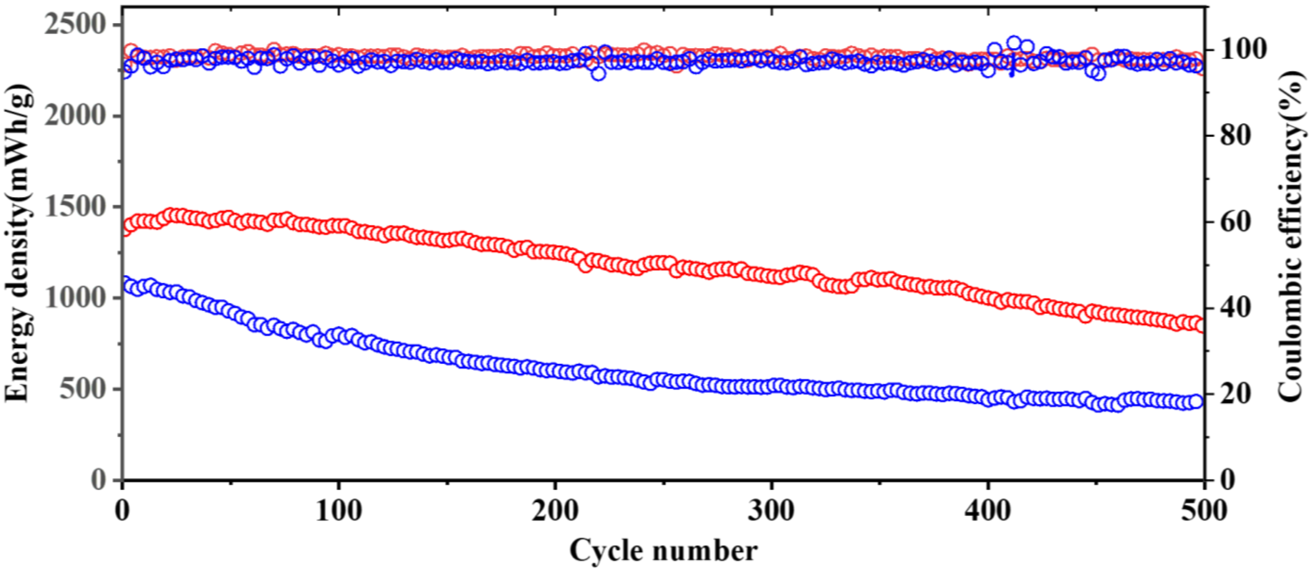

Figure S10. Energy density performance of PEO-based battery at 1 C (60 °C).

Table S1. Ionic conductivity results of polymer electrolyte (Unit: $S{\cdot}cm^{-1}$).

Temperature	PEO/LiTFSI	PEO-5%HP@TFSI	PEO-10%HP@TFSI	PEO-20%HP@TFSI
30 °C	4.7×10^{-6}	$5.7{\times}10^{-6}$	$6.1{\times}10^{-6}$	$3.0{\times}10^{-6}$
40 °C	1.7×10^{-5}	1.8×10^{-5}	2.3×10^{-5}	1.2×10^{-5}
50 °C	6.3×10^{-5}	4.3×10^{-5}	7.6×10^{-5}	5.5×10^{-5}
60 °C	3.0×10^{-4}	3.1×10^{-4}	3.3×10^{-4}	4.0×10^{-4}
70 °C	4.7×10^{-4}	5.2×10^{-4}	5.2×10^{-4}	6.2×10^{-4}
80 °C	8.1×10^{-4}	8.5×10^{-4}	7.6×10^{-4}	9.7×10^{-4}
90 °C	1.1×10^{-3}	1.1×10^{-3}	1.0×10^{-3}	1.4×10^{-3}

Table S2. Data of mechanical properties number of the four polymer electrolytes.

Electrolytes	Tensile Strength/MPa	Percentage of Breaking Elongation/%
PEO/LiTFSI	0.95	2549.4
PEO-5%HP@TFSI	1.24	2570.5
PEO-10%HP@TFSI	1.41	3677.19
PEO-20%HP@TFSI	1.45	4533.82

Table S3. Datas of Li-S batteries using different type of PEO-based electrolytes reported in literature.

Ref.	Cathode Composition	Electrolyte			Cycling Performance /mAh·g⁻¹
		Composition	IONIC Conductivity/S·cm⁻¹	Li+ Transference Number	
**This work**	**S/@C (6/4, 90 wt%) PVDF (10 wt%)**	**LiTFSI/PEO-20%HP@TFSI**	**9.7×10^{-4} (80 °C)**	**0.521**	**1400(1st cycle) 972(50th cycle) (60 °C, 0.1 C)**
1	S (40 wt%) Carbon (15 wt%) Electrolyte (45 wt%)	PEO/LiTFSI-$Li_{10}SnP_2S_{12}$	1.69×10^{-4} (50 °C)	0.38	1016 (1st cycle) 1000 (40th cycle) (60 °C, 0.1 C)
2	PEO (15 wt%) LiTFSI (5 wt%)	PEO/LITFSI-10%MMT	3.22×10^{-4} (25 °C)	0.45	998 (1st cycle) 634 (100th cycle) (60 °C, 0.1 C)
3	S (30.1 wt%)	PEO/LiTFSI-10%HNT	1.1×10^{-4} (60 °C)	0.4	809 (1st cycle) 386 (400th cycle) (100 °C, 4 C)
4	S (40 wt%) Carbon (15 wt%) Electrolyte (45 wt%)	PEO/LiTFSI	6.5×10^{-4} (70 °C)	/	1394 (1st cycle) 800 (60th cycle) (70 °C, 0.1 C)
5	S/@C (6/4, 90 wt%) PVDF (10 wt%)	PEO/LiTFSI-10%iCP@TFSI	1.2×10^{-3} (80 °C)	0.29	1237 (1st cycle) 1083 (100th cycle) (60 °C, 0.2 C)
6	S (40 wt%) Carbon (15 wt%) Electrolyte (45 wt%)	LiFSI/PEO	9×10^{-5} (70 °C)	0.12	800 (1st cycle) (70 °C, 0.05 C)
7	S/@C (7/3, 80 wt%) Carbon (10 wt%) CMC (10 wt%)	PEO/LiFTFSI-5%TiO_2	/	/	1450 (1st cycle) 1261 (100th cycle) (60 °C, 0.1 C)

Reference

1. Li, X.; Wang, D.; Wang, H.; Yan, H.; Gong, Z.; Yang, Y. Poly (ethylene oxide)-$Li_{10}SnP_2S_{12}$ composite polymer electrolyte enables high-performance all-solid-state lithium sulfur battery. *ACS Appl. Mater. Interfaces* **2019**, *11*, 22745–22753.
2. Zhang, Y.; Zhao, Y.; Gosselink, D.; Chen, P. Synthesis of poly(ethylene-oxide)/nanoclay solid polymer electrolyte for all solid-state lithium/sulfur battery. *Ionics* **2015**, *21*, 381–385.
3. Lin, Y.; Wang, X.; Liu, J.; Miller, J.D. Natural halloysite nano-clay electrolyte for advanced all-solid-state lithium-sulfur batteries. *Nano Energy* **2017**, *31*, 478–485.
4. Eshetu, G.G.; Judez, X.; Li, C.; Martinez-Ibañez, M.; Gracia, I.; Bondarchuk, O.; Armand, M. Ultrahigh performance all solid-state lithium-sulfur batteries: salt anion's chemistry-induced anomalous synergistic effect. *J. Am. Chem. Soc.* **2018**, *140*, 9921–9933.
5. Wang, Y.; Ji, H.F.; Shi, J.J.; Li, X.N.; Zhang, X.J.; Qu, X.W. Cyclopropenium Cationic-Based Covalent Organic Polymer Enhanced Polyethylene Oxide Composite Polymer Electrolyte for All-Solid-State Li-S Battery. *ACS Appl. Mater. Interfaces* **2021**, *13*, 16469–16477.

6. Judez, X.; Zhang, H.; Li, C.; González-Marcos, J.A.; Zhou, Z.; Armand, M.; Rodriguez-Martinez, L.M. Lithium bis (fluorosulfonyl) imide/poly (ethylene oxide) polymer electrolyte for all solid-state Li-S cell. *J. Phy. Chem. Lett*. **2017**, *8*, 1956–1960.
7. Lee, F.; Tsai, M.C.; Lin, M.H.; Ni'mah, Y.L.; Hy, S.; Kuo, C.Y.; Hwang, B.J. Capacity retention of lithium sulfur batteries enhanced with nano-sized TiO_2-embedded polyethylene oxide. *J. Mater. Chem. A* **2017**, *5*, 6708–6715.
